# Importance of early diagnosis and surgical treatment of calcified amorphous tumor-related native valve endocarditis caused by *Escherichia coli*: a case report

**DOI:** 10.1186/s12879-022-07220-w

**Published:** 2022-03-07

**Authors:** Shu Koito, Yuto Unoki, Keimei Yoshida, Sho Takemoto, Takayuki Uchida, Takashi Matono

**Affiliations:** 1 Department of General Internal Medicine, Aso Iizuka Hospital, Iizuka, Japan; 2 Department of Cardiology, Aso Iizuka Hospital, Iizuka, Japan; 3 Department of Cardiovascular Surgery, Aso Iizuka Hospital, Iizuka, Japan; 4 Department of Infectious Diseases, Aso Iizuka Hospital, 3-83 Yoshio, Iizuka, Fukuoka 820-8505 Japan

**Keywords:** Non-HACEK Gram-negative bacilli, *Escherichia coli*, Endocarditis, Calcified amorphous tumor

## Abstract

**Background:**

Unlike *Escherichia coli* bacteremia, which is common, *E. coli* endocarditis is uncommon, particularly in patients with native valve, leading to its delayed diagnosis.

**Case presentation:**

We present a case of infective endocarditis caused by *E. coli* in a 78-year-old Japanese man with type 2 diabetes, involving persistent bacteremia and vegetation on the mitral valve (measuring 18 × 4.2 mm in diameter). He presented with recurrent fever after antimicrobial treatment for pyelonephritis. He received antibiotic therapy for 6 weeks and required surgical removal of a calcified amorphous tumor and vegetation with mitral valvuloplasty 7 days after admission. Despite an episode of multiple cerebral infarctions, he recovered fully from the infection.

**Conclusions:**

Follow-up blood cultures should be performed for Gram-negative bacilli bacteremia among patients with unknown focus and an atypical clinical course after treatment. Early diagnosis and aggressive surgical intervention are paramount to achieving good clinical outcomes.

## Background

Gram-negative bacilli bacteremia is frequently encountered in clinical practice, accounting for approximately 50% of all bacteremia cases [1]. Specifically, *E. coli* is the most common causative pathogen in community-acquired infections [2], and the predominant sources of infection are the urinary tract, biliary tract, and other intra-abdominal infections. Occasionally, *E. coli* can cause potentially fatal infections, such as infected aortic aneurysms, vertebral osteomyelitis, and infective endocarditis. Non-HACEK (*Haemophilus parainfluenzae/aphrphilus*, *Actinobacillus actinomycetemcomitans*, *Cardiobacterium hominis*, *Eikenella corrodens*, and *Kingella* spp.) Gram-negative bacillus endocarditis is generally associated with endovascular devices and prosthetic heart valves; its incidence constitutes 1.8–5.0% of all endocarditis cases, with *E. coli* accounting for only 0.51% of all causative pathogens [3–5].

*E. coli* is one of the causative pathogens of non-HACEK Gram-negative bacillus endocarditis; however, *E. coli* endocarditis accounts for approximately 0.2% of *E. coli* bacteremia [6, 7]. A clinical practice dilemma exists regarding the necessity of performing follow-up blood cultures after a diagnosis of Gram-negative bacilli bacteremia. In most cases, these are not performed, which can substantially delay the diagnosis of complications, such as endocarditis. Reportedly, 90% of non-HACEK Gram-negative bacillus endocarditis cases were diagnosed > 1 month after the onset of symptoms. Consequently, the mortality rate of non-HACEK Gram-negative bacillus endocarditis (24%) is higher than that of endocarditis caused by other pathogens (17%) [3]. Herein, we report a rare case of an older adult who presented with calcified amorphous tumor-associated native valve endocarditis caused by *E. coli* and survived after an early diagnosis and aggressive surgical intervention.

## Case presentation

A 78-year-old Japanese man with ischemic heart disease, chronic heart failure, and type 2 diabetes was presented with a complaint of fever for 2 days. He had reportedly completed a 12-day course of intravenous ceftriaxone treatment for *E. coli* pyelonephritis without bacteremia, 8 days earlier. He had persistent fever despite having received empirical oral levofloxacin for the last 2 days and was admitted for further investigations and management. Physical examination revealed a body temperature of 38.9 °C, respiratory rate of 24 breaths per minute, heart rate of 116 beats per minute, and tenderness of the prostate gland. Laboratory findings showed a white blood cell count of 10,810/μL (neutrophils, 90.1%); C-reactive protein, 316 mg/L; hemoglobin A1c, 9.5%; urinary leukocytes, 0–1/HPF; and no evidence of urinary nitrites.

**Fig. 1:**
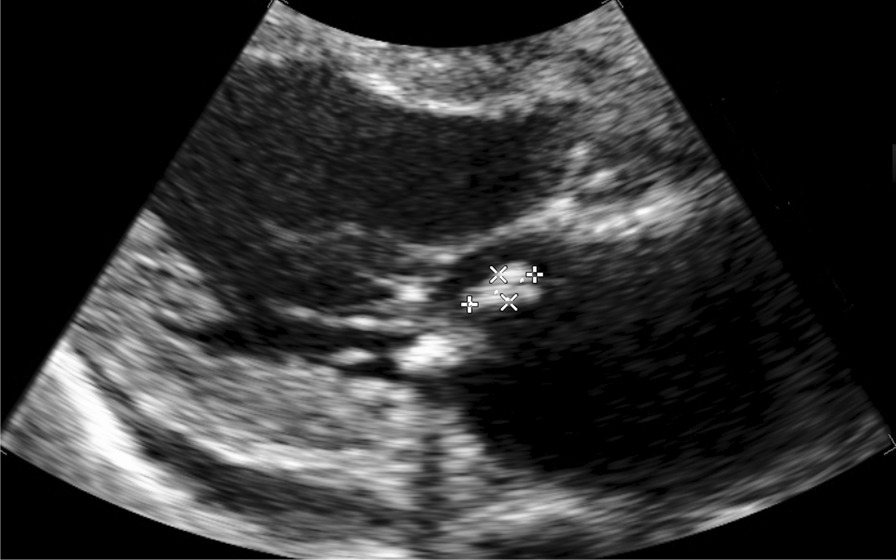
Transthoracic echocardiography performed on the day after admission. A hyperechoic mobile vegetation (measuring 13 × 5 mm in diameter) is detected on the P3 segment of the mitral valve, which shows annular calcification

**Fig. 2:**
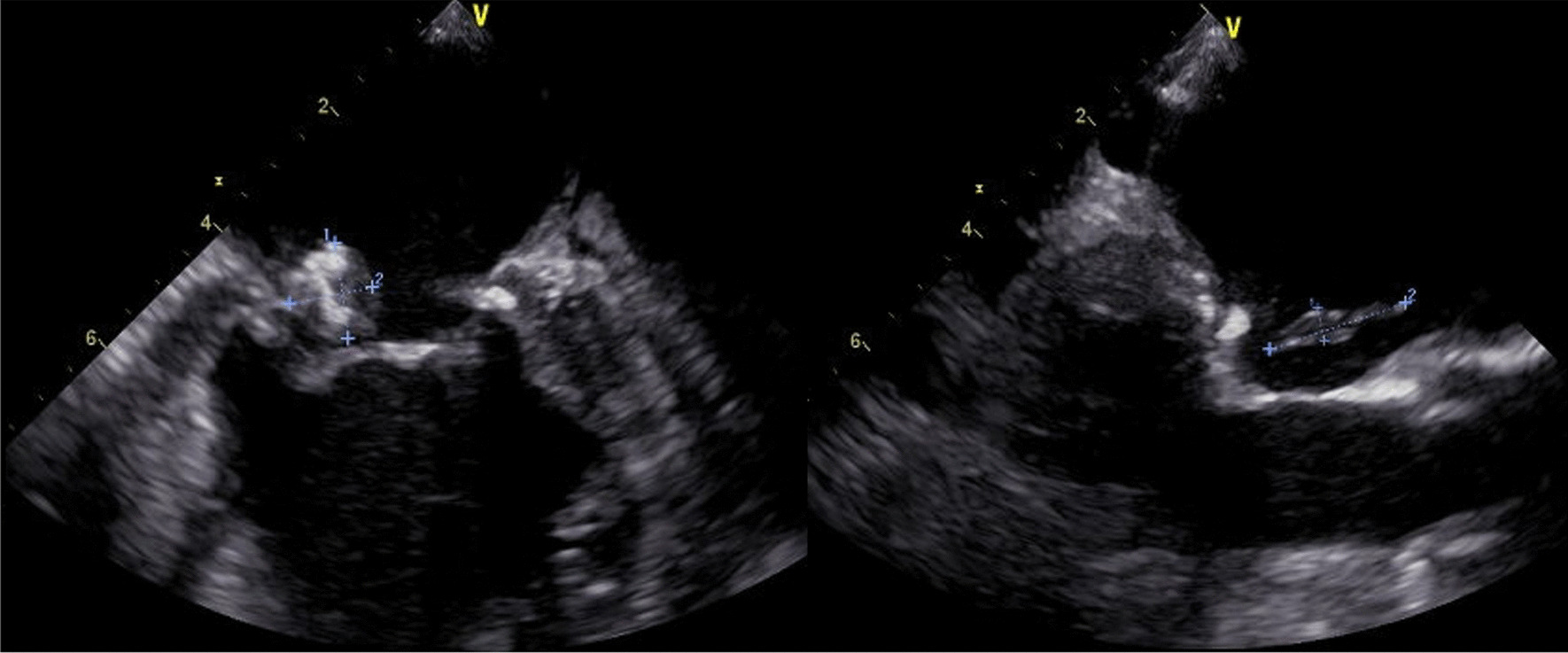
Transesophageal echocardiography performed 5 days after admission. A hyperechoic and hypoechoic calcified amorphous tumor and an isoechoic to hypoechoic mobile vegetation (measuring 18 mm × 4.2 mm in diameter, overall) are detected on the posterior mitral valve leaflet in both P2 and P3 regions

**Fig. 3:**
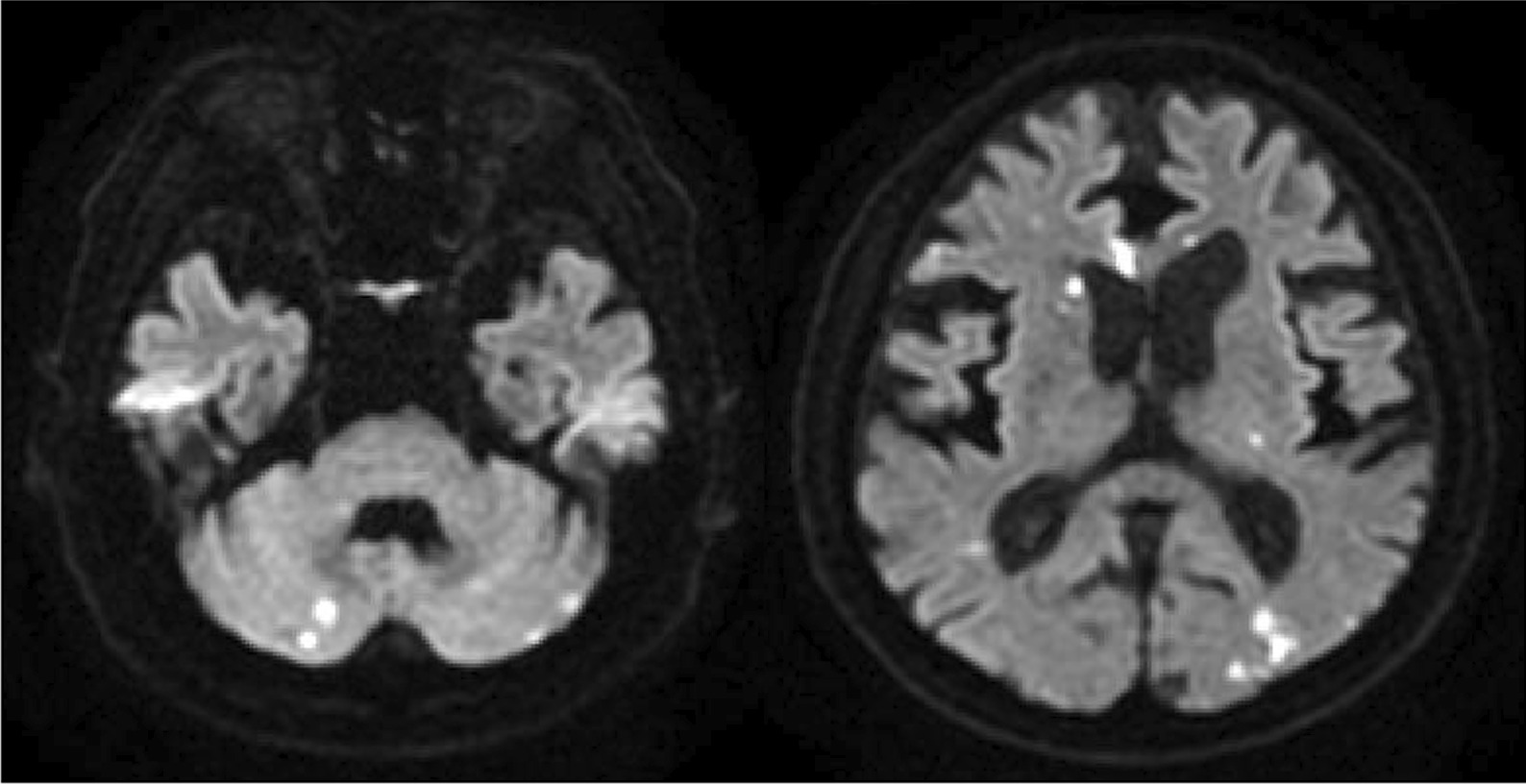
Cranial magnetic resonance imaging performed 4 days after admission. Bilateral acute multiple cerebral infractions are observed

**Fig. 4:**
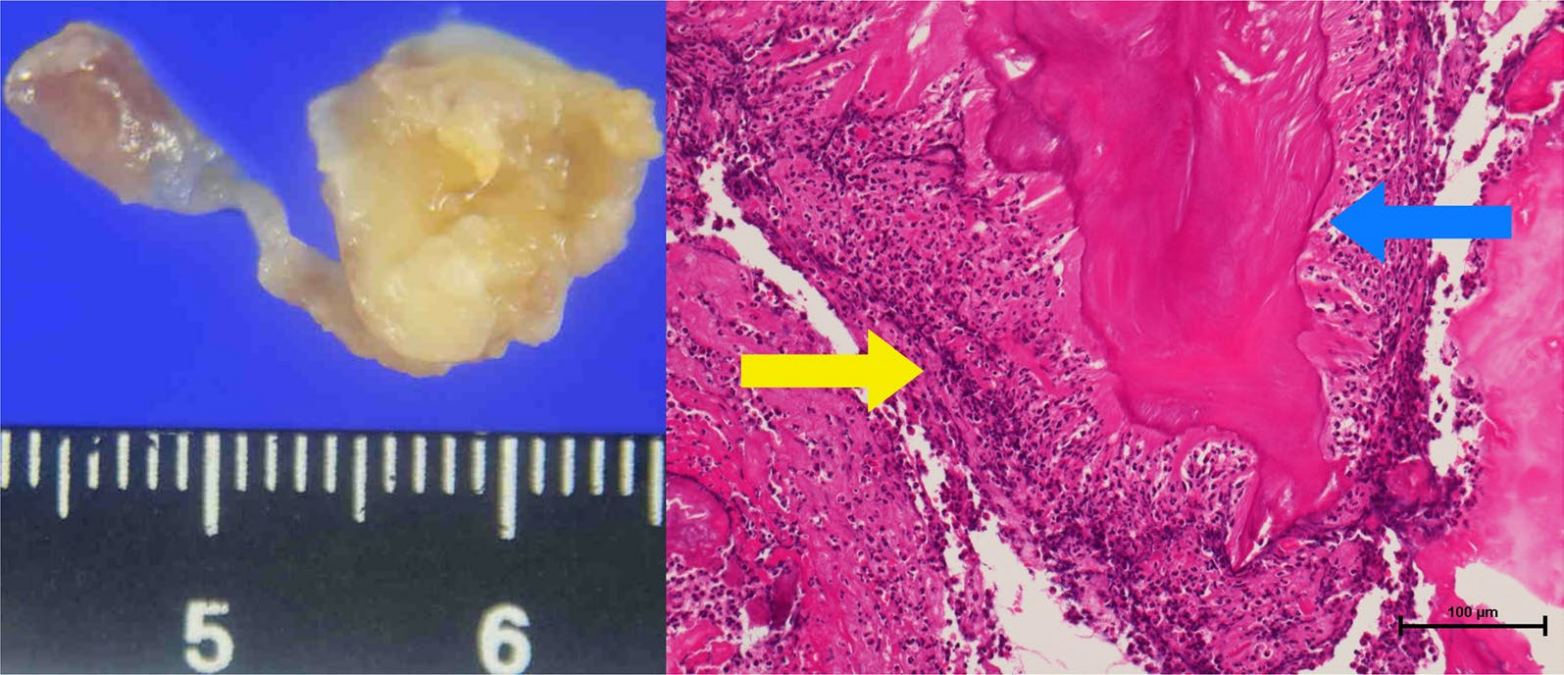
Vegetation on the mitral valve, which includes the calcified amorphous (blue arrow) and infiltration of neutrophils (yellow arrow)

A full-body enhanced computed tomography scan showed no evidence of infectious foci, including abscesses. However, blood culture results revealed the presence of *E. coli* bacteremia. Therefore, we initiated intravenous cefmetazole, considering suspected bacteremic prostatitis. However, transthoracic echocardiography was performed as part of the workup for recurrent fever, which revealed moderate mitral regurgitation and a high echoic structure (measuring 13 mm × 5 mm diameter) on the posterior mitral leaflet (Fig. 1). Since the structure was not detected in the previous transthoracic echocardiography performed 3-months earlier, we also conducted transesophageal echocardiography. It revealed a high and low echoic heterogenous immobilized structure (measuring 10.2 mm × 11.7 mm in diameter) attached to the annulus of the mitral valve from P2 to P3 and showed hypermobile vegetation (measuring 18 mm × 4.2 mm in diameter) attached continuously to the immobilized structure (Fig. 2). With bacteremia evidence and echocardiography findings, we diagnosed the patient with infective endocarditis associated with a suspicious calcified amorphous tumor. Antibiotic treatment was modified to ceftriaxone and gentamicin following the diagnosis because *E. coli* was not a multidrug-resistant strain. *E. coli* was detected on blood culture samples taken on day-1 and day-5 of hospitalization, confirming persistent bacteremia. On day-6 of hospitalization, the patient developed altered mental status. A head magnetic resonance imaging scan was performed, which revealed bilateral acute multiple cerebral infarctions (Fig. 3). Since no acute hemorrhagic transformation after the stroke was identified by the follow-up computed tomography, we performed removal of the vegetation and mitral valvuloplasty on day-7 of hospitalization. Cardiopulmonary bypass was established with the initial intravenous administration of heparin (300 U/kg) and maintained the activated clotting time above 400 s. Intravenous heparin was switched to oral warfarin postoperatively. There was no intraoperative or postoperative cerebral hemorrhage. Histopathological examination of the specimen showed calcified deposits and inflammatory granulation tissue infiltrated by neutrophils (Fig. 4). The patient experienced renal insufficiency 21 days after admission, and the treatment was changed from ceftriaxone with gentamicin to ceftriaxone with ciprofloxacin. Antibiotic treatment was administered for 6 weeks from the day of the first negative blood culture result (7 days after admission). The patient recovered fully from the infection and was transferred to a rehabilitation hospital 77 days after admission.

## Discussion and conclusion

We reported a rare case of community-acquired calcified amorphous tumor-related native valve *E. coli* endocarditis. While non-HACEK Gram-negative bacillus endocarditis is difficult to diagnose and often fatal, the patient survived the infection despite advanced age. Herein, we discuss potential causes of this favorable prognosis, with a focus on diagnosis and treatment.

First, we were able to establish the diagnosis of rare non-HACEK Gram-negative bacillus endocarditis from among other common microbial causes of Gram-negative bacilli bacteremia observed in clinical settings. Recent studies have indicated that the incidence of non-HACEK Gram-negative bacillus endocarditis has increased, particularly in older adults [8, 9], and the most common source is a urinary tract infection [10]. We believe follow-up cultures could aid the diagnostic process, as they would allow the detection of persistent bacteremia and warrant conducting subsequent transthoracic echocardiography. A review article suggested that follow-up blood cultures should be considered in patients with risk factors, including endovascular infection, bacteremia caused by *Staphylococcus aureus* and *Candida spp.*, infection due to a multidrug-resistant strain, febrile neutropenia, central venous catheter infection, treatment non-response, and an unknown source of infection [11, 12]. However, the yield of follow-up blood cultures is low (5.7%) in bacteremia caused by Gram-negative bacilli [13]. Consequently, clinical practices among health institutions regarding the collection and examination of follow-up blood culture samples for diagnosis of Gram-negative bacilli bacteremia are variable. Moreover, several risk factors for non-HACEK Gram-negative bacillus endocarditis have been identified, including central venous catheters, cardiac devices, history of heart disease, immunocompromised host status, old age, and concurrent diabetes [4, 9]. As our patient presented with several of these risk factors (old age, history of heart disease, and diabetes), we opted to perform a follow-up blood culture and conduct a transthoracic echocardiography screening, which enabled early diagnosis of native valve endocarditis. Therefore, we believe that, in patients with bacteremia caused by common Gram-negative bacilli, follow-up blood cultures and transthoracic echocardiography should be performed when the source of infection is unknown, risk factors are present, and there is an unusual clinical course (including recurrent fever) to ensure an early diagnosis of endocarditis. However, the sensitivity of transthoracic echocardiography for detecting valve vegetation is lower (50–70%) than that of transesophageal echocardiography (> 90%) [14, 15]. Therefore, in a high-risk patient who shows an unusual clinical course (including recurrent fever) together with continuous bacteremia of unknown origin caused by common Gram-negative bacilli, transesophageal echocardiography should be considered even if no vegetation is detected by transthoracic echocardiography.

Second, we examined factors responsible for the complete recovery of the patient. A case report revealed that the in-hospital mortality rate of *E. coli* endocarditis was similar to that of other common pathogens [16]. However, other studies have reported in-hospital mortality rates of non-HACEK Gram-negative bacillus endocarditis, including *E. coli,* as high as 13.8–24% due to diagnostic delay [3, 4, 6, 17, 18]. However, our patient experienced a complete recovery from the infection. An early diagnosis might be partly responsible for this. He was diagnosed 20 days after the initial onset of symptoms and 5 days after the relapse of fever, which is earlier than that reported previously (> 1 month latency) [3]. Furthermore, the importance of corrective cardiac surgery in non-HACEK Gram-negative bacillus endocarditis has been suggested by several authors [3, 6]. It is noteworthy that we could perform mitral valvuloplasty without valve replacement 7 days after admission. Furthermore, Falcone et al. showed that the presence of a multidrug-resistant strain was the predominant poor prognostic factor for non-HACEK Gram-negative bacillus endocarditis [4]. In the present case, the *E. coli* identified was not a multidrug-resistant strain and was, therefore, sensitive to the initial empirical antibiotic treatment. Hence, we believe that an early diagnosis, prompt surgical intervention, and a causal non-multidrug-resistant strain of *E. coli* contributed to this patient’s favorable outcome.

Third, we investigated the etiology of endocarditis, with particular focus on the factors causing the development of a calcified amorphous tumor. Our patient presented with a calcified amorphous tumor associated with the endocardial vegetation. A calcified amorphous tumor was first reported by Reynolds et al. in 1997 as a non-neoplastic intracardiac mass, which on histopathologic examination showed calcified nodules with degenerated blood components, chronic inflammatory changes, and fibrin-like deposits [19]. Generally, surgical resection is recommended because the calcified amorphous tumor may cause systemic embolisms, including cerebral infarctions and cardiac conduction disturbances, which may lead to sudden death [20]. Our case is noteworthy because the association between endocarditis and calcified amorphous tumors is extremely rare, and only one such case has been reported to date, caused by *Enterococci* [5]. Further studies are required to elucidate the causal relationship between endocarditis and the development of calcified amorphous tumors. Although we did not conduct molecular analysis to determine the virulence of the *E. coli* strain in this case, causative strains that cause endocarditis are thought to be extraintestinal pathogenic strains often belonging to group B2 and capable of invading the cardiac endothelium [21]. We speculate that in our patient, inadequate duration of treatment of suspected prostatitis may have induced *E. coli* endocarditis. Thus, calcified amorphous tumor presence and that of a highly pathogenic strain may have contributed to the development of endocarditis.

In conclusion, we reported the case of a 78-year-old man with *E. coli* endocarditis, which was associated with a calcified amorphous tumor that was successfully treated by surgery. This is an extremely rare example of an association between endocarditis and calcified amorphous tumors. Furthermore, this case allows us to recognize that follow-up blood cultures should be performed in patients with Gram-negative bacilli bacteremia of unknown origin and in those demonstrating an atypical clinical course after treatment, to detect rare but potentially fatal cases of persistent bacteremia and its complications. Early diagnosis of endocarditis is vital to administer the appropriate antimicrobials and ensure timely surgical interventions if warranted, which can lead to better outcomes.

## Data Availability

All data described in the manuscript are available upon request to the corresponding author.

## Abbreviations

HACEK: *Haemophilus parainfluenzae*/*aphrphilus*, *Actinobacillus actinomycetemcomitans*, *Cardiobacterium hominis*, *Eikenella corrodens*, and *Kingella* spp.
